# SAC-Optimized Fuzzy Variable Admittance Control for Lead-Through Teaching of Collaborative Robots

**DOI:** 10.3390/s26113576

**Published:** 2026-06-04

**Authors:** Yu Song, Guoqing Ma

**Affiliations:** 1 School of Mechatronic Engineering, Changchun University of Science and Technology, Changchun 130022, China; 2023100594@mails.cust.edu.cn; 2 Chongqing Research Institute, Changchun University of Science and Technology, Chongqing 401133, China

**Keywords:** collaborative robot, lead-through teaching, variable admittance control, fuzzy control, soft actor-critic

## Abstract

In collaborative robot lead-through teaching, fixed admittance parameters impose an inherent trade-off between operational ease and motion stability. This paper proposes a SAC-optimized fuzzy variable admittance control method (SAC-FAC). A fuzzy variable admittance controller (FAC) quantifies the operator’s motion and turning intents using interaction-force and end-effector motion information, and modulates the damping coefficient online via interpretable fuzzy rules. Soft Actor-Critic (SAC) searches offline for a well-balanced membership-function configuration on an episode basis in simulation, and the optimized configuration is then fixed for deployment. A saturation mechanism in the reward function suppresses degeneration of the damping configuration toward its physical lower bound. To counter parameter degradation under high-disturbance training, potential-based reward shaping and performance-gated curriculum learning are jointly introduced to promote stable convergence. Ablation studies and comparisons with four alternative optimizers verify the training design and support the suitability of SAC in this framework. Experiments on a UR10 collaborative robot platform with three trajectory types show that, relative to the hand-tuned FAC, SAC-FAC reduces the mean trajectory tracking error, work per unit path, and root-mean-square interaction force by 19.5%, 11.6%, and 6.8%, respectively, with more evident advantages on the compound and 3D ramp trajectories while preserving the interpretability of the fuzzy rule structure.

## 1. Introduction

Lead-through teaching allows an operator to record motion trajectories by directly guiding the end-effector of a collaborative robot, and has been increasingly used in flexible manufacturing owing to its intuitive interaction and flexible deployment [1]. It is generally implemented through two approaches: one estimates interaction forces from robot dynamic models or joint currents, while the other measures them directly using force/torque sensors mounted at the end-effector [2]. The latter provides direct multi-axis force information for intent-oriented compliance control. In sensor-based lead-through teaching, the measured interaction forces are converted into end-effector motion through an admittance control model [3], in which the damping coefficient is the key compliance parameter. However, fixed damping creates an inherent trade-off: a low damping value produces high responsiveness but provides insufficient suppression of minor disturbances, which can lead to trajectory deviations, whereas a high damping value improves motion stability at the cost of increased operator effort. This contradiction has motivated research on variable admittance control, in which admittance parameters are adjusted online according to operator intent [4].

Beyond this performance trade-off, interaction-force regulation during lead-through teaching is also related to the current safety framework for industrial robot applications. The updated ISO 10218-1:2025 and ISO 10218-2:2025 standards [5,6] provide safety requirements for industrial robots and their applications, while ISO/TS 15066:2016 [7] provides dedicated requirements and guidance for collaborative industrial robot systems. For hand-guided lead-through teaching, these standards establish a safety context in which interaction forces should be considered during system design and risk assessment. Thus, adaptive regulation of interaction forces is relevant not only to teaching quality, but also to safety considerations in collaborative operation.

Existing variable admittance methods have advanced along two main directions: rule-based or fuzzy-inference parameter adjustment and data-driven automatic tuning. Hamad et al. [8] designed a fuzzy logic controller that adjusts admittance gains online based on interaction force and velocity, demonstrating the feasibility of fuzzy methods for variable admittance. Kang et al. [9] introduced intent recognition into variable admittance control by adjusting admittance parameters according to operator intent categories. To reduce reliance on explicit rules, Sharkawy et al. [10] employed a neural network to learn operator interaction patterns online for admittance adjustment, though at the expense of interpretability compared with fuzzy approaches. Han et al. [11] proposed a variable admittance controller based on an energy cost function with gradient-descent-based parameter tuning, illustrating the data-driven trend in this field. Among these approaches, fuzzy inference remains an important design paradigm because its interpretable rule structure provides transparent and traceable damping adjustment logic, which is particularly valuable in human-in-the-loop tasks such as lead-through teaching. Nevertheless, the tuning of membership function parameters still relies on manual experience, making it difficult to obtain a well-balanced configuration across competing objectives such as translational responsiveness and braking stability.

Reinforcement Learning (RL) offers a trial-and-error-based learning framework for automatic optimization of variable admittance parameters. In early explorations, Dimeas and Aspragathos [12] and Du et al. [13] applied fuzzy Q-learning and fuzzy Sarsa(λ), respectively, to online damping adjustment in variable admittance control, with the latter validated on a single-degree-of-freedom surgical manipulator. However, both methods were constrained by the discrete state–action spaces inherent to tabular RL, limiting their scalability to high-dimensional continuous problems. With the development of Deep Reinforcement Learning (DRL), optimization has been extended to continuous spaces, and several studies have adopted algorithms such as Deep Deterministic Policy Gradient (DDPG) and Actor-Critic for online optimization of variable admittance or impedance parameters [14,15,16]. Soft Actor-Critic (SAC) has also been employed to learn variable impedance gains and to optimize controller parameters offline [17,18,19]. However, these DRL-only studies did not incorporate a fuzzy inference structure. Recent studies have combined DRL with fuzzy logic in peg-in-hole assembly, where fuzzy modules were used to generate impedance baselines or assist reward design [20,21]. Both works, however, target assembly scenarios and adopt deterministic policy algorithms, with fuzzy logic serving an auxiliary role rather than constituting the core controller structure. Furthermore, Han et al. [22] embedded SAC policy gradients into a fuzzy neural network to update PD controller membership function parameters online, demonstrating the feasibility of SAC-based fuzzy parameter optimization. However, their end-to-end coupling precludes separation of SAC and the fuzzy controller at deployment, and validation was limited to simulation benchmarks. Compared with structured assembly, lead-through teaching involves more time-varying and less predictable operator intent. The use of SAC as a standalone offline optimizer for fuzzy variable admittance controller parameters, followed by real-robot deployment, has not been systematically investigated.

Building on the research gap identified above, this paper proposes a SAC-optimized fuzzy variable admittance control method (SAC-FAC). The maximum-entropy framework of SAC encourages exploratory search [23]. In this work, this property is used to search for membership-function configurations that remain effective across the scheduled force-amplitude conditions in simulation. Based on force/torque sensing, this paper defines intent factors to quantify operator dragging intent and designs a Fuzzy Variable Admittance Controller (FAC) for intent-adaptive damping modulation. On this basis, SAC is employed to optimize the membership function parameters offline in a simulation environment; upon completion, the parameters are fixed for deployment on the real robot, with only fuzzy inference and admittance control executed during the online phase. The suitability of SAC is assessed through comparisons with DDPG, TD3, PPO, and Bayesian Optimization (BO), and the training mechanisms are examined through ablation studies. Lead-through teaching experiments with three trajectory types on a UR10 collaborative robot equipped with an ATI Axia80 six-axis force/torque sensor demonstrate that SAC-FAC outperforms the hand-tuned FAC in both trajectory tracking accuracy and operating force cost.

The remainder of this paper is organized as follows. Section 2 presents the admittance model, operator intent factors, fuzzy variable admittance controller, and SAC-based offline optimization framework. Section 3 evaluates the suitability of SAC through optimizer comparison and verifies the necessity of the training mechanisms through ablation studies. Section 4 reports the real-robot lead-through teaching experiments on the UR10 platform, and Section 5 discusses the experimental results and methodological implications. Section 6 concludes the paper and outlines future work.

## 2. System Overview and Methods

**Figure 1:**
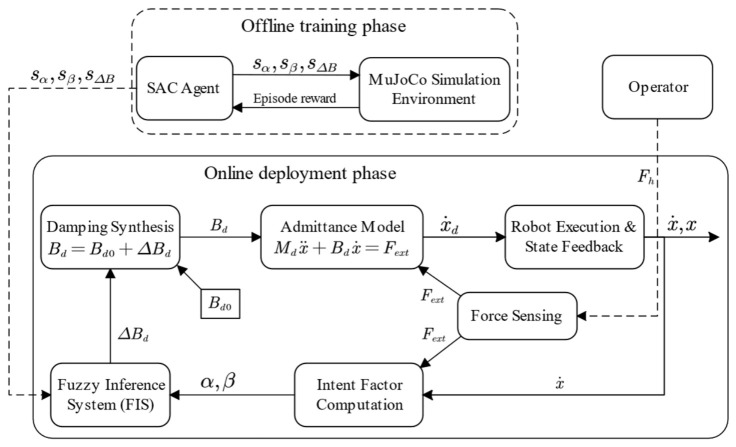
Architecture of the proposed SAC-FAC framework.

The proposed SAC-FAC framework comprises an offline training phase and an online deployment phase, as illustrated in Figure 1. This section first establishes the admittance model and the operator intent factors, then designs the fuzzy variable admittance controller and the teaching performance indices, and finally presents the SAC-based offline optimization framework for membership function parameters.

### 2.1. Admittance Modeling and Operator Intent Quantification

During lead-through teaching, the operator–robot interaction force is measured in real time by a six-axis force/torque sensor mounted at the end-effector. Because the raw force signal is affected by sampling noise and zero drift, a Kalman filter [24] is used for recursive estimation. The prediction and update steps are:

(1) $$\left\{\begin{array}{c} {\bar{X}}_{k}=A\cdot {\hat{X}}_{k-1}+B\cdot u_{k-1},{\bar{P}}_{k}=A\cdot {\hat{P}}_{k-1}\cdot A^{⊤}+Q\\ K_{k}={\bar{P}}_{k}\cdot H^{⊤}(H\cdot {\bar{P}}_{k}\cdot H^{⊤}+R{)}^{-1}\\ {\hat{X}}_{k}={\bar{X}}_{k}+K_{k}(Y_{k}-H\cdot {\bar{X}}_{k}),{\hat{P}}_{k}={\bar{P}}_{k}-K_{k}\cdot H\cdot {\bar{P}}_{k} \end{array}\right.$$

where A is the state transition matrix, while B and $u_{k-1}$ denote the input matrix and control input in the general form, respectively. In the force-signal filtering used in this work, no explicit control input is applied. H is the observation matrix, Q and R are the process and measurement-noise covariance matrices, and $K_{k}$ is the Kalman gain. Building on the filtered signal, a multi-pose least-squares calibration method [25] is adopted to establish a gravity compensation model that removes the gravitational contribution of the end-effector payload in real time according to the current robot pose, thereby extracting the external interaction force:

(2) $$F_{\mathrm{ext},i}=F_{i}-F_{i0}-G_{i}(R)$$

where $F_{i}$ is the raw measurement on the *i*-th sensor axis, $F_{i0}$ is the zero-point offset of that axis, and $G_{i}\left(R\right)$ is the gravitational component of the end-effector payload projected onto the *i*-th sensor axis according to the current rotation matrix R. Details of the experimental platform configuration and signal-processing parameters are provided in Section 4.1.

Admittance control achieves compliant interaction by establishing a virtual mechanical relationship between external forces and end-effector motion [3]. Its general form is:

(3) $$K_{d}\cdot (x-x_{0})+B_{d}\cdot (\dot{x}-{\dot{x}}_{0})+M_{d}\cdot (\ddot{x}-{\ddot{x}}_{0})=F_{ext}$$

where $M_{d}$, $B_{d}$, and $K_{d}$ are the virtual mass, damping, and stiffness matrices, respectively; x denotes the end-effector position; and $x_{0}$ denotes the reference position. In lead-through teaching, the stiffness term generates a restoring force that impedes free dragging, while the teaching trajectory is determined directly by the operator rather than by a predefined reference. Therefore, $K_{d}$ is set to zero to eliminate the restoring force, and the model is described in an incremental form so that the reference position term is omitted. Because both $M_{d}$ and $B_{d}$ are diagonal matrices, the axes are decoupled, and the single-degree-of-freedom admittance equation reduces to:

(4) $$m_{d}\ddot{x}+b_{d}\dot{x}=f_{\mathrm{ext}}$$

Setting $\ddot{x}=0$ yields the steady-state velocity ${\dot{x}}_{ss}=f_{ext}/b_{d}$, which depends solely on the damping coefficient; the time constant $\tau =m_{d}/b_{d}$ is governed by both mass and damping. These relationships indicate that $B_{d}$ is the dominant parameter for regulating lead-through compliance. Following the guideline in [26] that recommends a virtual mass-to-damping ratio of approximately 1:10, this study sets $M_{d}=5$ kg and treats $B_{d}$ as the parameter to be adjusted.

To enable the admittance system to modulate the damping coefficient adaptively according to operator dragging intent, a quantitative description of the intent is required. This study fuses velocity and force information to construct an intent direction vector d:

(5) $$d=\frac{v+\lambda F_{ext}}{|v+\lambda F_{ext}|+\varepsilon }$$

where v is the end-effector velocity vector, $F_{\mathrm{ext}}$ is the external interaction force vector, λ is a dimensional scaling coefficient with units of m/(N·s), and ε is a small positive constant on the order of 10^−6^ to prevent division by zero. In this study, λ was fixed at 1.0 × 10^−3^ m/(N·s). This small dimensional scaling coefficient is not a tunable control parameter; it keeps $\lambda \left|F_{\mathrm{ext}}\right|$ sufficiently greater than ε during start-up while keeping $\lambda \left|F_{\mathrm{ext}}\right|$ small relative to $\left|v\right|$ during normal lead-through motion. On this basis, the motion intent factor α is defined as the projection of the interaction force onto the intent direction:

(6) $$\alpha =F_{\mathrm{ext}}\cdot d=F_{\mathrm{ext}}\cdot \frac{v+\lambda F_{\mathrm{ext}}}{|v+\lambda F_{\mathrm{ext}}|+\varepsilon }$$

When the robot is in motion and $\left|v\right|\gg \lambda \left|F_{\mathrm{ext}}\right|$, d approximately aligns with the velocity direction, and α approximates the tangential component of the interaction force along the velocity. When $\left|v\right|$ approaches zero and $\lambda \left|F_{\mathrm{ext}}\right|$ is much larger than ε, d reduces to the force direction, and α approximates the force magnitude. This formulation allows α to characterize operator intent consistently under both moving and near-stationary conditions. For the UR10 collaborative robot used in the real-robot experiments, which has a rated payload of 10 kg, the physical range of α is set to [−15, 15] N to describe the signed propulsion/braking intent under moderate manual guidance. This interval covers the push–pull interaction forces considered in this study while avoiding an unnecessarily broad input range for subsequent fuzzification. Positive values indicate acceleration intent, whereas negative values indicate braking intent.

The turning intent factor β quantifies the operator’s intent to change the direction of motion and is defined as the normal component of the interaction force relative to the velocity direction:

(7) $$\beta =\frac{\left|F_{\mathrm{ext}}\times v\right|}{|v|+\varepsilon }\approx |F_{\mathrm{ext}}|\sin\theta$$

where θ is the angle between the interaction force and the velocity, and the approximation holds when $\left|v\right|\gg \varepsilon$. Because β is defined as the magnitude of the normal component of the interaction force, it is non-negative by construction and geometrically bounded by $\left|F_{\mathrm{ext}}\right|$. Its physical range is set to [0, 10] N to describe lateral steering effort for turning-intent discrimination, rather than the main tangential force associated with propulsion or braking. This compact range helps preserve the sensitivity of the subsequent fuzzy partitions to weak and moderate steering inputs.

Based on the combined values of α and β, the lead-through teaching process can be decomposed into six representative stages. As listed in Table 1, this decomposition provides the direct basis for the subsequent design of the fuzzy rule base.

### 2.2. Fuzzy Variable Admittance Controller

Building on the admittance model and intent factors established in Section 2.1, a dual-input, single-output fuzzy variable admittance controller is designed to adaptively modulate the damping coefficient $B_{d}$ online. The controller takes the motion intent factor α and the turning intent factor β as inputs and produces the damping increment $\Delta B_{d}$ as its output. The final damping coefficient is synthesized as:

(8) $$B_{d}=B_{d0}+\Delta B_{d}$$

where $B_{d0}$ is the baseline damping coefficient. Following the virtual mass $M_{d}=5$ kg determined in Section 2.1 and the recommended mass-to-damping ratio of approximately 1:10, $B_{d0}$ is set to 50 N·s/m. The physical range of $\Delta B_{d}$ is defined as $\left[-30,30\right]$ N·s/m, yielding an effective damping range of $B_{d}\in \left[20,80\right]$ N·s/m, which balances responsiveness at low damping with motion stability and acceptable operating effort at high damping.

**Figure 2:**
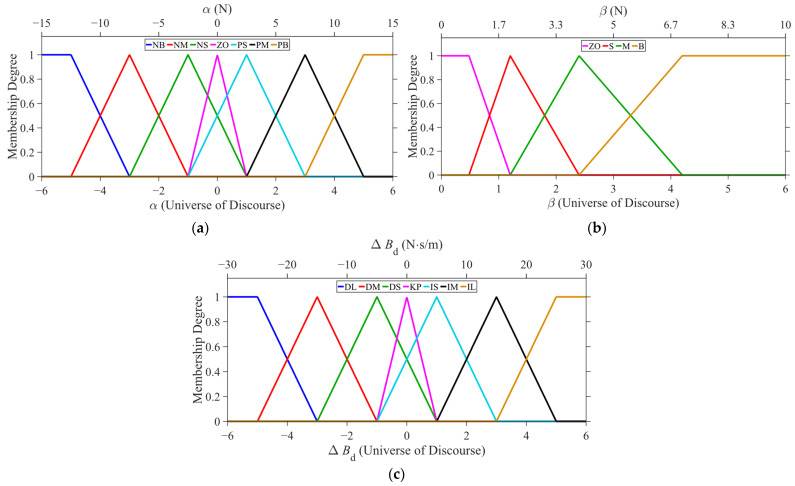
Membership functions of the fuzzy variable admittance controller: (**a**) motion intent factor α; (**b**) turning intent factor β; (**c**) damping increment $\Delta B_{d}$.

Seven linguistic variables are assigned to α to describe motion intent from strong deceleration to strong acceleration: NB (Negative Big), NM (Negative Medium), NS (Negative Small), ZO (Zero), PS (Positive Small), PM (Positive Medium), and PB (Positive Big). Four linguistic variables are assigned to β, namely ZO (Zero), S (Small), M (Medium), and B (Big), because β represents only the magnitude of turning intent. The output $\Delta B_{d}$ is described by seven linguistic variables: DL (Decrease Large), DM (Decrease Medium), DS (Decrease Small), KP (Keep), IS (Increase Small), IM (Increase Medium), and IL (Increase Large). The physical ranges of α, β, and $\Delta B_{d}$ are mapped to the universes of discourse [−6, 6], [0, 6], and [−6, 6], yielding the input quantization factors $K_{\alpha }=0.4$ N^−1^ and $K_{\beta }=0.6$ N^−1^, and the output scaling factor $K_{B}=5$. The corresponding membership functions are shown in Figure 2.

Triangular and trapezoidal membership functions are adopted because their breakpoints have explicit physical meanings and require only simple interpolation during inference. Trapezoidal shoulder functions are used at the boundaries to maintain full activation over the extreme linguistic states, whereas triangular functions are used in the intermediate regions to provide continuous transitions between adjacent states. This piecewise-linear structure is suitable for real-time fuzzy reasoning and for the subsequent low-dimensional SAC-based scaling of membership-function breakpoints, while preserving the interpretability of the rule base.

Based on the six-stage intent characteristics and damping adjustment directions summarized in Table 1, a fuzzy rule base containing 28 rules is constructed, as presented in Table 2. Each entry in the table denotes the output linguistic label of $\Delta B_{d}$ for the corresponding combination of α and β. The controller employs Mamdani-type inference [27] for rule activation and aggregation, followed by centroid defuzzification to convert the fuzzy output into a crisp damping increment $\Delta B_{d}$.

To quantitatively evaluate the compliance performance of the fuzzy variable admittance control system in lead-through teaching tasks, the interaction force $F_{\mathrm{ext}}$ is decomposed into a tangential component $F_{t}$ and a normal component $F_{n}$ with respect to the direction of end-effector motion. Two evaluation indices are defined according to the ratio between motion output and force cost: the translational compliance index $I_{t}$ and the rotational compliance index $I_{n}$.

The translational compliance index $I_{t}$ evaluates compliance performance under straight-line propulsion conditions and is defined as the ratio of the path length traversed by the end-effector to the cumulative tangential force exerted by the operator:

(9) $$I_{t}=\frac{\int_{0}^{T}vdt}{\int_{0}^{T}\left|F_{t}\right|dt+\varepsilon }$$

where T is the duration of the applied force, v is the end-effector speed, $\left|F_{t}\right|$ is the absolute tangential force component used to prevent sign cancellation in alternating push–pull motions, and ε is a small positive constant on the order of 10^−6^. The unit of $I_{t}$ is m/(N·s); a higher value indicates a greater path response per unit tangential-force cost.

The rotational compliance index $I_{n}$ evaluates turning performance under curved-trajectory conditions and is defined as the ratio of the cumulative change in motion direction to the normal force cost along the trajectory:

(10) $$I_{n}=\frac{\Delta \theta }{\int_{0}^{L}\left|F_{n}\right|ds+\varepsilon }$$

where Δθ is the total angle swept by the velocity vector over the entire motion, L is the total arc length of the trajectory, ds is the arc-length differential, and $\left|F_{n}\right|$ is the absolute normal force component. The unit of $I_{n}$ is rad/(N·m); a higher value indicates a greater directional change per unit normal-force cost. These two indices provide the quantitative basis for comparing FAC and SAC-FAC and for the reward function design in Section 2.3.

### 2.3. SAC-Based Optimization of Membership Function Parameters

The FAC designed in Section 2.2 performs intent-adaptive damping modulation through fuzzy inference, but its membership-function parameters still rely on manual tuning. The tuning process is challenging: the propulsion mode requires lower damping to reduce operator effort, whereas turning and force-release stages require higher damping to preserve motion stability. To address this issue, membership-function tuning is formulated as an episode-level MDP, and SAC is used offline in simulation to search for a balanced fixed-parameter configuration. After training, the optimized parameters are deployed as constants; online control consists only of force-signal processing, fuzzy inference, and admittance control, without invoking the SAC policy network online [23].

#### 2.3.1. Episode-Level Optimization Architecture and MDP Formulation

Because the optimized parameters remain fixed during real-robot deployment, an episode-level scaling-factor optimization scheme is adopted. At the beginning of each training episode, SAC outputs a triplet of scaling factors $\left(s_{\alpha },s_{\beta },s_{\Delta B}\right)$ that rescale the membership-function breakpoints for α, β, and $\Delta B_{d}$. These factors remain fixed throughout the episode. The simulator then executes a complete force-interaction episode and returns the cumulative reward. This design aligns the training objective with fixed-parameter deployment and reduces the action space to three scaling factors, helping the algorithm converge within a limited training budget.

The state representation is defined as a one-dimensional scalar, $\eta \in \left[0.5,1.2\right]$ denoting the disturbance level scheduled by the curriculum learning mechanism. This level determines the amplitude range of the external force signal in simulation, with larger η corresponding to stronger interaction forces. Because SAC observes only η, rather than the force waveform or velocity response within an episode, it is encouraged to learn scaling-factor combinations that remain robust across different interaction conditions.

The action is defined as a three-dimensional continuous vector. The SAC policy network outputs $a={\left[a_{\alpha },a_{\beta },a_{\Delta B}]\in [-1,1\right]}^{3}$, which is mapped linearly to the feasible ranges of the scaling factors:

(11) $$s_{\alpha }=0.97+0.03\cdot a_{\alpha },\;s_{\alpha }\in [0.94,1.00]$$

(12) $$s_{\beta }=0.85+0.35\cdot a_{\beta },\;s_{\beta }\in [0.50,1.20]$$

(13) $$s_{\Delta B}=1.20+0.40\cdot a_{\Delta B},\;s_{\Delta B}\in [0.80,1.60]$$

Because the quantization and scaling factors defined in Section 2.2 establish a bijective mapping between the physical ranges and the corresponding universes of discourse, all breakpoints are defined and manipulated directly in the physical domain without affecting the fuzzy inference result. To preserve the original coverage of the input and output ranges, only the non-boundary breakpoints of the membership functions are rescaled, while the boundary points remain fixed. Specifically, the non-boundary breakpoints of the input membership functions for α and β are rescaled by $s_{\alpha }$ and $s_{\beta }$, respectively. For the output membership functions of $\Delta B_{d}$, the non-boundary breakpoints are scaled with $\Delta B_{d}=0$ N·s/m as the symmetry center:

(14) $$bp_{new}=bp_{base}\times s_{\Delta B}$$

Since $bp_{base}$ is expressed as an offset from $\Delta B_{d}=0$, multiplication by $s_{\Delta B}$ scales the breakpoint symmetrically about the center. This mechanism ensures that the boundary trapezoidal functions (NB/PB for α, ZO/B for β, and DL/IL for $\Delta B_{d}$) always cover the full physical range, preventing activation gaps in the rule base. For numerical implementation, centroid defuzzification is approximated using 101 equally spaced discrete points over $\Delta B_{d}\in \left[-30,30\right]$ N·s/m.

#### 2.3.2. Task Rewards and Scaling-Factor Constraints

The reward function has four components. The propulsion reward $r_{push}$ and turning reward $r_{turn}$ are adapted from the compliance indices $I_{t}$ and $I_{n}$ defined in Section 2.2. The potential-based reward shaping term $r_{PBRS}$ is derived from the kinetic energy of the admittance system, whereas the conditional stability reward $r_{cond}$ penalizes residual velocity during the release stage. Because $I_{t}$ and $I_{n}$ are trajectory-level indices, their “motion output/force cost” ratio structure is recast into per-step reward terms, with a saturation mechanism introduced to prevent degenerate damping reduction.

The propulsion reward is defined as:

(15) $$r_{push}=40\cdot \frac{\tanh\left(\left\|\dot{x}\right\|/v_{ref}\right)}{\max\left(\left|F_{t}\right|,1\right)}$$

where $\left|F_{t}\right|$ denotes the absolute tangential force component, consistent with the denominator of Equation (9). The clipping term $\max\left(\left|F_{t}\right|,1\right)$ imposes a lower bound of 1 N on the denominator to avoid unbounded reward values under near-zero force. The numerator uses a tanh saturation term rather than a raw velocity term: a reward linear in velocity would drive SAC to reduce damping unconditionally, causing the fuzzy controller to degenerate into a constant minimum-damping controller. As the velocity approaches $v_{ref}$, the tanh term saturates and suppresses the incentive for further damping reduction. The reference velocity is set to $v_{ref}=0.40$ m/s, corresponding to the steady-state speed of the hand-tuned FAC under the maximum propulsion input used in the training simulation: when $\alpha_{max}\approx 11.3$ N and the corresponding damping is approximately 28.5 N·s/m, $v_{ss}=\alpha_{max}/B_{d}\approx 0.40$ m/s.

The turning reward is also adapted from $I_{n}$ and is activated only when the turning intent is sufficiently clear:

(16) $$r_{turn}={\dot{\theta }}_{v}\cdot I\left(\beta >0.5\right)$$

where ${\dot{\theta }}_{v}\geq 0$ denotes the magnitude of the velocity-direction change rate between adjacent time steps, and $I\left(\cdot \right)$ is the indicator function. The threshold β>0.5 N ensures that the reward is activated only under explicit turning intent, avoiding spurious turning rewards caused by force-signal noise during straight-line propulsion.

Although the tanh saturation suppresses the bias toward minimum damping, SAC may still exploit persistently low damping to maximize $r_{push}$ while neglecting release-stage braking. A conditional stability reward is therefore introduced to penalize residual velocity after force release:

(17) $$r_{cond}=-\sigma \left(\left\|F_{ext}\right\|\right)\cdot \mu \cdot {\left\|\dot{x}\right\|}^{2}$$

(18) $$\sigma \left(\left\|F_{ext}\right\|\right)=\frac{1}{1+\exp\left(\frac{\left\|F_{ext}\right\|-F_{th}}{\tau_{\sigma }}\right)}$$

where $\sigma \left(\cdot \right)$ is a soft switching function based on the external force magnitude. The switch approaches zero during sustained force application and rises toward unity after release, thereby activating the velocity penalty only in the release phase. The release detection threshold is set to $F_{th}=1.0$ N, the smoothing parameter to $\tau_{\sigma }=0.5$ N, and the penalty strength to μ=30.0.

The variation in admittance-system kinetic energy is further used as a complementary shaping signal. According to the policy invariance theorem of Ng [28], a potential-based reward shaping term can provide additional learning guidance while preserving the optimal policy set of the original MDP. In this work, the negative kinetic energy of the admittance system is selected as the potential function:

(19) $$\Phi (s)=-V(s)=-\frac{1}{2}M_{d}{\left\|\dot{x}\right\|}^{2}$$

Substituting Equation (19) into the PBRS shaping formula and applying a scaling coefficient yields the shaping term:

(20) $$r_{PBRS}=c_{pbrs}\left(\frac{1}{2}M_{d}{\left\|{\dot{x}}_{t}\right\|}^{2}-\gamma \cdot \frac{1}{2}M_{d}{\left\|{\dot{x}}_{t+1}\right\|}^{2}\right)$$

where γ=0.99 is the discount factor. The term $r_{PBRS}$ reflects the change in system kinetic energy between adjacent time steps, and $c_{pbrs}$ scales the shaping-signal magnitude. The total reward is defined as:

(21) $$r_{total}=w_{t}\cdot r_{push}+w_{n}\cdot r_{turn}+\lambda_{1}\cdot r_{PBRS}+\lambda_{2}\cdot r_{cond}$$

where $r_{push}$ and $r_{turn}$ are task rewards, $r_{PBRS}$ is the potential-based shaping term, and $r_{cond}$ is a release-stage constraint term. Because $\lambda_{1}=1.0$, $r_{PBRS}$ preserves the policy-invariant shaping form; $r_{cond}$ is not policy-invariant and is activated only after force release to constrain residual motion. The weights are assigned according to their roles: $w_{t}=1.0$ for the primary propulsion objective, $w_{n}=0.5$ because turning episodes account for a smaller portion of training while still requiring directional sensitivity, $\lambda_{1}=1.0$ for shaping, and $\lambda_{2}=0.5$ to penalize residual motion without dominating the task rewards.

The saturation mechanism suppresses damping reduction at the reward level, but it cannot by itself prevent degeneration of the membership-function shapes. Physical constraints are therefore imposed on the scaling factors in the action space. For α, the PB trapezoidal membership function has a rising edge from 7.5 N to 12.5 N; after scaling, these breakpoints become $7.5\times s_{\alpha }$ and $12.5\times s_{\alpha }$. If $s_{\alpha }$ is too small, $\alpha_{max}\approx 11.31$ N falls inside the PB plateau, where the membership degree is identically 1, eliminating the controller’s ability to distinguish higher force amplitudes. Requiring the PB membership degree at $\alpha_{max}$ to be no greater than $\mu^{*}=0.90$ gives:

(22) $$s_{\alpha }\geq \frac{\alpha_{max}}{7.5+5\;\mu^{*}}$$

Substituting $\alpha_{max}=11.31$ N and $\mu^{*}=0.90$ gives $s_{\alpha }\geq 0.94$; therefore, the feasible range is set to $s_{\alpha }\in \left[0.94,\;1.00\right]$. The ranges $s_{\beta }\in \left[0.50,\;1.20\right]$ and $s_{\Delta B}\in \left[0.80,\;1.60\right]$ provide sufficient freedom for compressing or expanding the *β* and $\Delta B_{d}$ membership functions while preventing severe shape degeneration.

#### 2.3.3. Performance-Gated Curriculum Learning and Training Configuration

A performance-gated curriculum learning mechanism is adopted to improve controller robustness over a range of external force amplitudes. Unlike fixed-schedule curricula [29], the proposed mechanism updates the disturbance level according to recent episode returns: the level is upgraded once the calibrated threshold is reached and downgraded when the return falls below the downgrade threshold. This avoids transitions that either advance too early or spend excessive training budget at easy levels.

The disturbance levels are defined as $\left\{\eta_{0},\eta_{1},\eta_{2},\eta_{3},\eta_{4}\}=\{0.50,0.65,0.80,1.00,1.20\right\}$, with training starting from the lowest level $\eta_{0}$. The disturbance level η scales the external force amplitude in the simulation environment, and the corresponding force ranges are listed in Table 3.

Upon entering a new level, level transitions are suspended for W=50 episodes, during which return data are collected for threshold calibration:

(23) $$R_{up}=0.85\cdot \frac{1}{W}\sum_{i=1}^{W}R_{i}$$

(24) $$R_{down}=0.6\cdot R_{up}$$

where $R_{i}$ is the return of the *i*-th calibration episode. The factor 0.85 provides a margin for return variability. The downgrade threshold is set at 60% of $R_{up}$, and a downgrade is triggered only when the recent return falls below $R_{down}$. After calibration, the average return ${\bar{R}}_{W}$ over the most recent W episodes is monitored, and the disturbance level is updated as follows:

(25) $$\mathrm{Upgrade}:\;{\bar{R}}_{W}\geq R_{up}\;\mathrm{and}\;l<4$$

(26) $$\mathrm{Downgrade}:\;{\bar{R}}_{W}<R_{down}\;\mathrm{and}\;l>0$$

The conditions l<4 and l>0 prevent further upgrades at the highest level and further downgrades at the lowest level, respectively. After each transition, a new 50-episode calibration window and a 10-episode cooldown period are applied before the next switching decision.

Training uses SAC [23] in the MuJoCo physics engine [30] with Stable Baselines3 (SB3) [31]. The main hyperparameters are summarized in Table 4. Each episode comprises 100 steps: 70 force-application steps followed by 30 release steps, during which the external force decays to zero and provides training signals for $r_{cond}$. Each episode randomly samples one of three force modes: propulsion 50%, turning 25%, and braking 25%.

In the subsequent optimizer comparison, each algorithm is independently run with multiple random seeds to assess training stability. The comparative evaluation of these algorithms, the ablation analysis of training-mechanism components, and the determination of the final deployment parameters are presented in Section 3.

## 3. Optimizer Selection and Ablation Analysis

This section evaluates the proposed optimization framework through optimizer selection and ablation analysis. Five optimization algorithms are first compared with five random seeds each to identify a suitable offline optimizer and a fixed-parameter deployment candidate. An ablation study is then conducted on the selected configuration to examine the necessity of potential-based reward shaping, curriculum learning, and performance gating.

### 3.1. Optimizer Comparison and Selection

**Figure 3:**
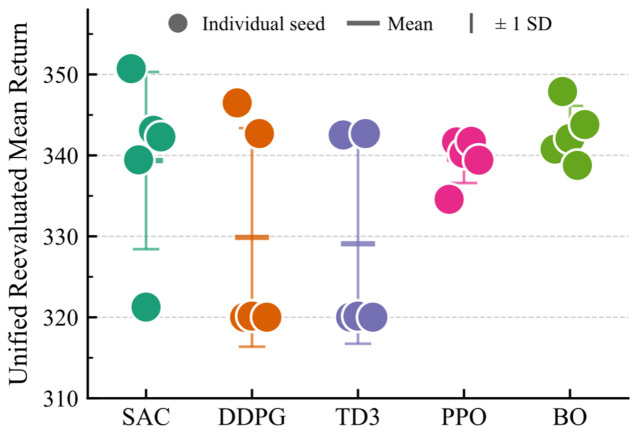
Unified reevaluated returns over five random seeds. Dots denote individual seed results, and bars indicate the mean ± sample standard deviation for each optimizer.

Five optimization algorithms—SAC, DDPG [32], TD3 [33], PPO [34], and BO [35]—are compared for fixed-parameter deployment under the same three-dimensional scaling-factor parameterization ($s_{\alpha }$, $s_{\beta }$, and $s_{\Delta B}$), reward function, and reevaluation protocol. The algorithm-specific sampling mechanisms and search budgets are retained: SAC, DDPG, and TD3 are each allotted 1000 episodes per seed, PPO is allotted 1024 timesteps per seed, and BO is allotted 25,000 environment episodes per seed. Each algorithm is run with five random seeds: 42, 43, 44, 45, and 46. After training, each optimized parameter set is reevaluated for five episodes at each of the five disturbance levels, and the average return is used as the reevaluated return for that seed. The results are shown in Figure 3 and Table 5.

A scaling factor is classified as a boundary parameter when its distance to either constraint bound is no greater than 1.0 × 10^−3^. The boundary count in Table 5 is calculated over five seeds × three scaling factors for each optimizer, giving 15 parameters in total, and thus characterizes the optimizer’s parameter distribution in the constrained search space. For DDPG and TD3, all 15 scaling factors are classified as boundary parameters, indicating a strong tendency to produce near-boundary solutions in this task. Although these two methods occasionally achieve high-return candidates, their relatively large seed-to-seed variation and concentration of parameters near the constraint boundaries make them less suitable for fixed-parameter deployment in this study.

BO achieves the highest five-seed mean return (342.7 ± 3.5), confirming its competitiveness in this low-dimensional offline optimization problem. Its training time is substantially longer than that of SAC, but this cost affects only offline retuning rather than online deployment, since the optimized parameters obtained by both methods are fixed during real-time control. Nevertheless, the best BO candidate has a lower return than the best SAC candidate (347.9 vs. 350.7), and two of its scaling factors are classified as boundary parameters. PPO shows the smallest return variation and no boundary parameters, but its best-seed return (341.7) is also lower than that of SAC. For SAC, seven boundary parameters are observed across the five seeds; however, its best-return candidate, seed 42, contains no boundary parameter and achieves the highest best-seed return among all optimizers. These observations indicate that the optimizer comparison should consider not only the five-seed mean return, but also the candidate-level return, boundary-parameter behavior, and offline training cost.

To further assess the local robustness of the best-return candidate selected from each optimizer, a ±5% single-dimensional perturbation is applied to each scaling factor while keeping the other two factors unchanged. Perturbations that violate the feasible range are excluded. The baseline return in Table 6 is the local-perturbation baseline and is used only for within-candidate comparison, rather than for direct comparison with the reevaluated returns in Table 5. A perturbation direction is regarded as an observable improvement when the return increase is greater than 0.5. The results are summarized in Table 6.

As shown in Table 6, the SAC candidate shows no observable improvement under the tested ±5% single-parameter perturbations, with a maximum return increase of only +0.23. DDPG, TD3, and PPO also show no observable improvement, but this result should be interpreted together with Table 5, where DDPG and TD3 exhibit boundary-dominated parameter distributions and PPO has a lower best-seed return than SAC. The BO candidate still has one observable improvement direction, with a maximum return increase of +0.51. Taken together, the five-seed reevaluation results, boundary-parameter behavior, local perturbation response, and offline training cost support the selection of SAC seed 42 as the fixed-parameter deployment candidate for the subsequent ablation study and real-robot experiments.

### 3.2. Ablation Study

**Figure 4:**
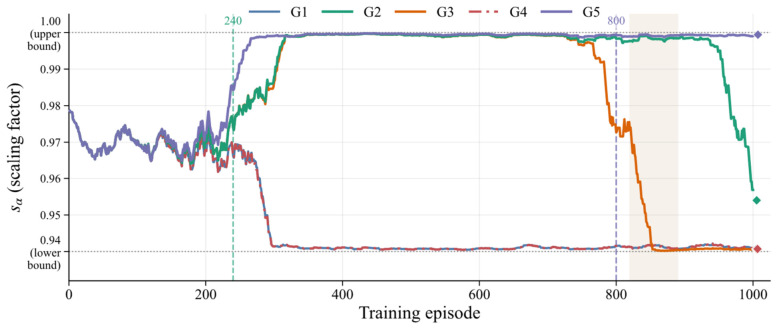
$s_{\alpha }$ training trajectories across the five ablation groups (seed = 42; smoothing window = 25).

Five ablation groups (G1–G5) are designed to examine the necessity of $r_{PBRS}$, curriculum learning, and performance gating. The purpose is not to compare different optimizers again, but to isolate how each training-mechanism component contributes to the final SAC-FAC parameter configuration. The groups differ in whether these components are enabled and in the resulting disturbance schedule, while all other training settings are kept unchanged. The configurations and results are summarized in Table 7, and the $s_{\alpha }$ training trajectories are shown in Figure 4.

The $s_{\alpha }$ values in Table 7 are deterministic policy outputs at the end of training and correspond to the diamond markers at episode 1000 in Figure 4; the σ (last-50) column reports the standard deviation of $s_{\alpha }$ over the final 50 training episodes. Only G2 produces an interior deterministic output ($s_{\alpha }$ = 0.9540), whereas the other four groups settle on the constraint boundary, with σ (last-50) ≤ 0.002 indicating little training-stage variation around the boundary. The larger σ of 0.027 for G2 reflects SAC’s continued maximum-entropy exploration around the interior solution, while its deterministic output at episode 1000 remains at 0.9540. The differences in $s_{\beta }$ and $s_{\Delta B}$ across groups remain small (Table 7).

G1 baseline: SAC without the three components cannot reach an interior solution. With $r_{PBRS}$, curriculum learning, and performance gating all disabled, $s_{\alpha }$ rapidly drops from its initial value to the lower bound of 0.9407 and remains there.

Necessity of $r_{PBRS}$ (G2 vs. G3): without $r_{PBRS}$, G3’s $s_{\alpha }$ also collapses to the lower bound (0.9405). As shown in Figure 4, $s_{\alpha }$ first reaches a near-upper-bound region and stays there for about 450 episodes, then collapses to the lower bound during episodes 820–890. In contrast to G1, which never explores the interior region, G3 reaches it but fails to stabilize there. This suggests that $r_{PBRS}$ mainly stabilizes an already discovered interior solution rather than promoting initial exploration.

Necessity of curriculum learning (G2 vs. G4): G4 enables $r_{PBRS}$ but trains entirely under a fixed η=1.0, yielding $s_{\alpha }=0.9407$—nearly identical to G1. The G1 and G4 curves in Figure 4 are almost superimposed. Under fixed η=1.0, $r_{PBRS}$ alone is too weak relative to the primary task reward $r_{push}$ to redirect the optimization. Curriculum learning first leads the parameters to a region where interior convergence is feasible at low disturbance levels, enabling $r_{PBRS}$ to take effect at higher levels.

Necessity of performance gating (G2 vs. G5): G5 includes both $r_{PBRS}$ and curriculum learning but uses a fixed schedule of 200 episodes per level, with $s_{\alpha }$ stalling at 0.9994 near the upper bound. As shown in Figure 4, G5 does not reach the highest disturbance level until episode 800, leaving only 200 episodes of training. Since the $r_{PBRS}$ signal is strongest at the highest level, this duration is insufficient for $s_{\alpha }$ to reach an interior solution. By contrast, G2’s performance gating adaptively allocates training episodes and preserves sufficient training time at the highest disturbance level.

In summary, the three components play distinct and complementary roles: $r_{PBRS}$ stabilizes the interior solution, curriculum learning enables $r_{PBRS}$ to influence the optimization under increasing disturbance levels, and performance gating prevents the training budget from being exhausted at lower levels. Their joint use is necessary for obtaining the non-boundary G2 configuration ($s_{\alpha }=0.9540$), which provides the deployment parameters analyzed in Section 3.3.

### 3.3. Deployed Membership Function Configuration

The deterministic policy outputs of the G2 configuration are adopted as the deployed membership function parameters of the fuzzy controller: $s_{\alpha }=0.954$, $s_{\beta }=0.508$, and $s_{\Delta B}=1.594$. These three scaling factors modify the membership-function breakpoints of the motion-intent input, the turning-intent input, and the damping-increment output, respectively.

**Figure 5:**
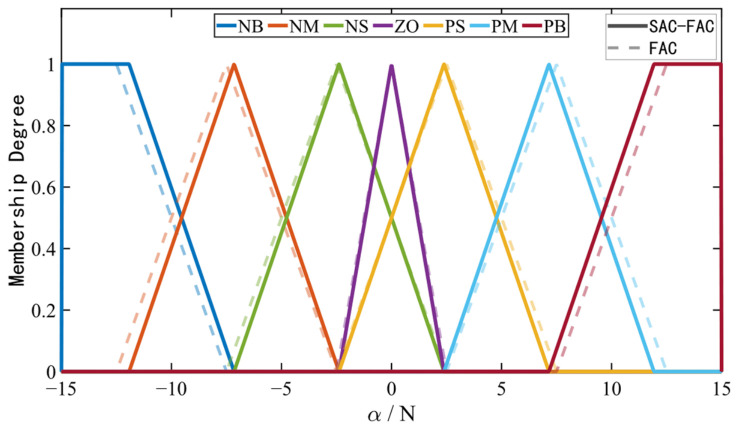
Comparison of α membership functions for FAC and SAC-FAC.

As shown in Figure 5, the α membership functions undergo the smallest shape change after SAC optimization. With $s_{\alpha }=0.954$, the non-boundary breakpoints contract by only about 4.6%, and the triangular and trapezoidal profiles nearly coincide before and after optimization. This indicates that, within the narrow constraint range [0.94, 1.00] derived in Section 2.3.2, SAC fine-tunes rather than restructures the original force-amplitude discrimination.

**Figure 6:**
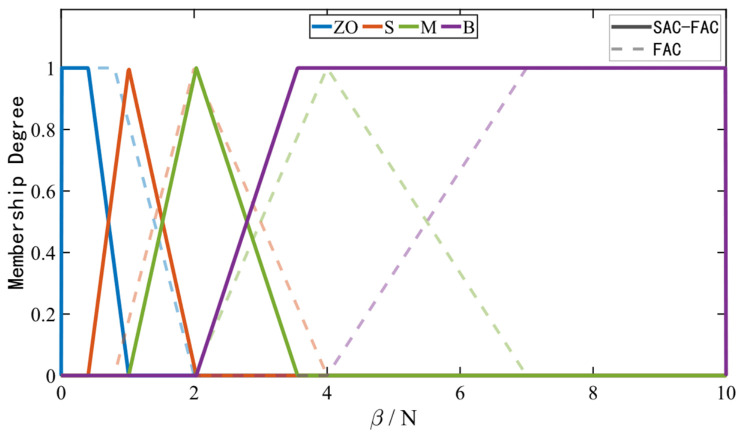
Comparison of β membership functions for FAC and SAC-FAC.

As shown in Figure 6, the β membership functions change most substantially. With $s_{\beta }=0.508$, all non-boundary breakpoints are compressed to about half of their initial positions, and the non-boundary breakpoint range spanning the transitions among the adjacent linguistic variables ZO, S, M, and B narrows from [0, 7] N to [0, 3.5] N. Taking the B (Big) trapezoid membership function as an example, its plateau onset shifts from about 7 N to 3.5 N, substantially lowering the activation threshold for turning-related rules.

**Figure 7:**
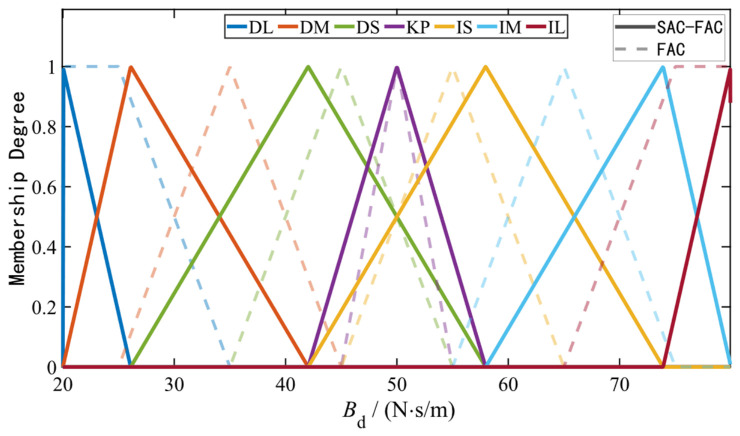
Comparison of output membership functions mapped onto the actual damping axis for FAC and SAC-FAC.

As shown in Figure 7, the optimized output membership functions are expressed on the actual damping axis. The non-boundary breakpoints of the $\Delta B_{d}$ membership functions expand outward from the baseline damping $B_{d0}=50$ N·s/m. Specifically, the peak of DM shifts from about 35 N·s/m to 27 N·s/m, while the peak of IM shifts from about 65 N·s/m to 74 N·s/m. The DL/DM region clusters toward the low-damping boundary (20 N·s/m), whereas the IM/IL region clusters toward the high-damping boundary (80 N·s/m). This outward expansion increases the magnitude of possible defuzzified damping corrections, thereby widening the effective damping-correction range of the controller.

Acting together, these complementary changes enhance turning sensitivity and broaden damping adjustment without altering the original fuzzy rule structure, thereby preserving the interpretability of the deployed controller. The deployed membership-function configuration is further evaluated in the physical lead-through teaching experiments in Section 4.

## 4. Lead-Through Teaching Experiments

Building on the SAC-FAC framework established in Section 2 and the optimizer selection and ablation results in Section 3, the optimized membership-function parameters are deployed on the real robot and evaluated through lead-through teaching experiments.

### 4.1. Experimental Platform and Gravity-Compensation Validation

**Figure 8:**
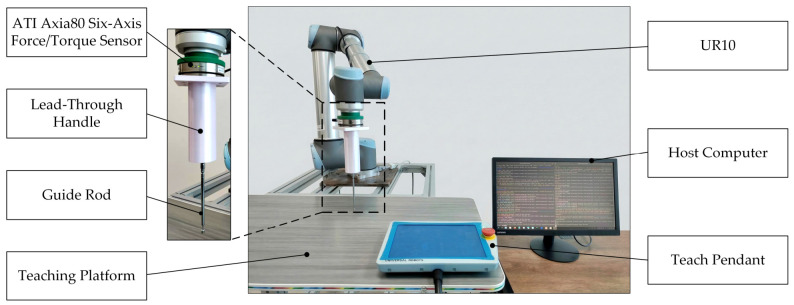
Lead-through teaching experimental platform.

The experimental platform is shown in Figure 8. It comprises a UR10 six-degree-of-freedom collaborative robot (Universal Robots A/S, Odense S, Denmark) with a 10 kg payload and a 1300 mm reach, an ATI Axia80 six-axis force/torque sensor (ATI Industrial Automation, Apex, NC, USA) with a 500 Hz sampling rate, a 3D-printed lead-through handle with a guide rod, a teaching platform, and a host computer. The force/torque sensor is mounted via a rigid flange between the robot end-flange and the handle. During the experiment, the operator grasps the handle to guide the end-effector along the desired path, while the guide-rod tip serves as a visual reference for deviations from the predefined trajectory.

The software system is built on Ubuntu 20.04 and ROS 2 Humble. Raw six-axis force/torque data are sampled at 500 Hz, filtered by a Kalman filter to suppress measurement noise, and processed by the gravity-compensation module described in Section 2.1 to obtain the external interaction force $F_{ext}$, which is then fed into the admittance control loop. The control loop runs at 125 Hz and supports three controller types: fixed admittance, FAC, and SAC-FAC. In the comparative experiments, the fixed-admittance controller is tested with two damping settings. Each controller produces joint velocity commands that are sent to the robot. During the experiments, the interaction force, end-effector pose and velocity, and damping coefficient are logged at 25 Hz for offline analysis.

For validation, the multi-pose least-squares calibration described in Section 2.1 was performed using six stationary poses with spatially distributed and mutually non-coplanar end-effector orientations. After compensation, the residual force magnitude at every validation pose was below 0.02 N, with a root-mean-square residual of 0.014 N. Relative to the pre-compensation mean force magnitude of 2.18 N, this corresponds to a 99.4% reduction, indicating sufficient force-signal accuracy for the subsequent lead-through teaching experiments.

### 4.2. Experimental Design

To compare different admittance control strategies, four configurations are set up, as listed in Table 8. All four share the same virtual mass $M_{d}=5$ kg, virtual stiffness $K_{d}=0$, and 125 Hz control rate, differing only in the damping setting. FAC and SAC-FAC are both fuzzy variable admittance controllers; the difference is that SAC-FAC uses membership function parameters obtained from offline SAC optimization. During online deployment, only fuzzy inference and admittance control are executed, and the SAC policy network is not involved.

**Figure 9:**
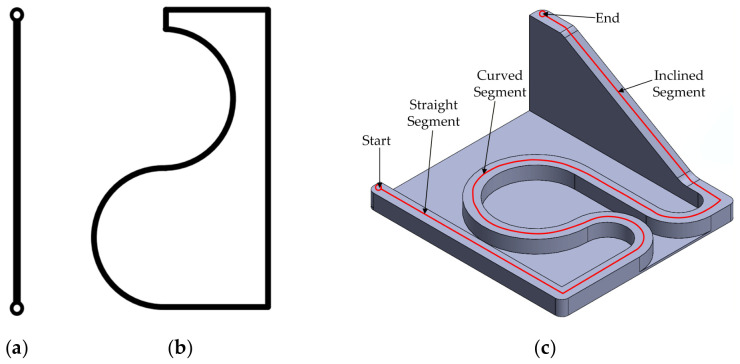
Reference trajectory designs: (**a**) straight-line; (**b**) compound; (**c**) 3D ramp.

Three reference trajectories of progressively increasing complexity are designed, as shown in Figure 9.

The straight-line trajectory (approximately 363 mm, XY plane) is used to evaluate compliance and tracking accuracy under pure-propulsion conditions. The compound trajectory (approximately 1196 mm, XY plane) is a closed path formed by concatenating straight and curved segments; it covers acceleration, constant-velocity, turning, and deceleration phases, and is used to examine controller response under frequent transitions in motion intent. The 3D ramp trajectory (approximately 1356 mm) consists of straight, curved, and 30–inclined segments, introducing a vertical displacement component for evaluation in a spatial lead-through task.

The reference for each trajectory is generated by programming a motion routine on the UR teach pendant. The guide-rod tip is driven along the path marked on the teaching platform, and the end-effector TCP pose is logged at 125 Hz through the Real-Time Data Exchange (RTDE) interface.

The interaction quality of lead-through teaching is evaluated in terms of trajectory accuracy, interaction-work cost, and interaction-force level, complemented by the compliance indices $I_{t}$ and $I_{n}$ defined in Section 2.2. The trajectory root-mean-square error $e_{RMSE}$, given in mm, is defined as:

(27) $$e_{RMSE}=\sqrt{\frac{1}{N}\sum_{i=1}^{N}d_{i}^{2}}$$

where $d_{i}$ is the shortest Euclidean distance from the end-effector position at the *i*-th sampling point to the reference trajectory, and N is the total number of samples. A smaller $e_{RMSE}$ indicates a closer match between the executed and reference trajectories.

The work per unit path $W_{u}$, given in N, characterizes the accumulated interaction work per unit travelled distance and is defined as:

(28) $$W_{u}=\frac{\int \left|F_{ext}\left(t\right)\cdot \dot{x}\left(t\right)\right|dt}{\int \left\|\dot{x}\left(t\right)\right\|dt}$$

where $F_{ext}\left(t\right)$ is the external interaction force vector, $\dot{x}\left(t\right)$ is the end-effector velocity vector, and the denominator represents the travelled path length. The single vertical bars around $F_{ext}\left(t\right)\cdot \dot{x}\left(t\right)$ denote the absolute value of the instantaneous interaction power, which prevents positive and negative work from canceling each other during acceleration and braking phases. Therefore, a smaller $W_{u}$ indicates a lower operating-force cost.

The root-mean-square interaction force $F_{RMS}$, given in N, characterizes the overall interaction-force level throughout the dragging process and is calculated as:

(29) $$F_{RMS}=\sqrt{\frac{1}{N}\sum_{i=1}^{N}\|F_{ext,i}{\|}^{2}}$$

where $F_{ext,i}$ is the sampled external interaction force vector at the *i*-th sampling point. A smaller $F_{RMS}$ indicates a lower overall interaction-force level.

$I_{t}$ is computed on the entire straight-line trajectory and on the straight segments of the compound and 3D ramp trajectories; $I_{n}$ is computed only on the curved segments of the latter two. For $e_{RMSE}$, $W_{u}$, and $F_{RMS}$, smaller values indicate better performance, whereas larger values of $I_{t}$ and $I_{n}$ indicate better compliance.

With the strategies, trajectories, and indicators specified, the 4 strategies × 3 trajectories yielded 12 experimental conditions. Each condition was repeated 50 times by the same operator to reduce operator-related variability, giving 600 trials in total. Outliers were removed using the 1.5 × IQR rule applied to $e_{RMSE}$, and the reported means and standard deviations were computed from the retained samples. To assess whether the improvements from FAC to SAC-FAC were statistically reliable, planned FAC-versus-SAC-FAC comparisons were conducted for each trajectory and metric using two-sided Welch’s *t*-tests on the retained samples. The two fixed-damping strategies were treated as reference baselines, and *p* < 0.05 was considered statistically significant. For the trajectory and time-series plots, the trial whose $e_{RMSE}$ was closest to the within-group mean was selected as the representative case, and the force and damping curves were smoothed using a 0.2 s moving average to suppress high-frequency fluctuations.

### 4.3. Results and Analysis

#### 4.3.1. Straight-Line Trajectory

**Figure 10:**
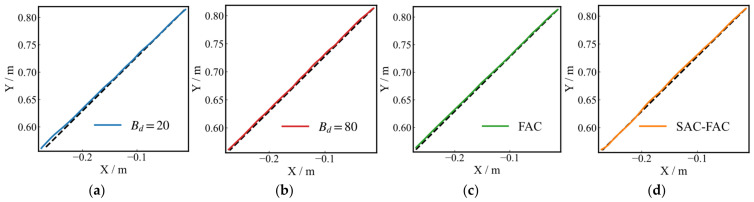
Straight-line dragging trajectories under four control strategies: (**a**) $B_{d}=20$ N·s/m; (**b**) $B_{d}=80$ N·s/m; (**c**) FAC; (**d**) SAC-FAC. Dashed lines: reference trajectory.

For the straight-line trajectory (β≈0), Figure 10 compares the dragging trajectories of the four strategies.

From Figure 10, the $B_{d}=20$ N·s/m strategy shows a larger lateral deviation in the middle segment, suggesting that low damping is insufficient to suppress minor operator-induced disturbances. The $B_{d}=80$ N·s/m strategy exhibits a smaller deviation, while FAC and SAC-FAC follow the reference trajectory more closely. Among them, SAC-FAC shows slightly tighter alignment with the reference trajectory.

**Figure 11:**
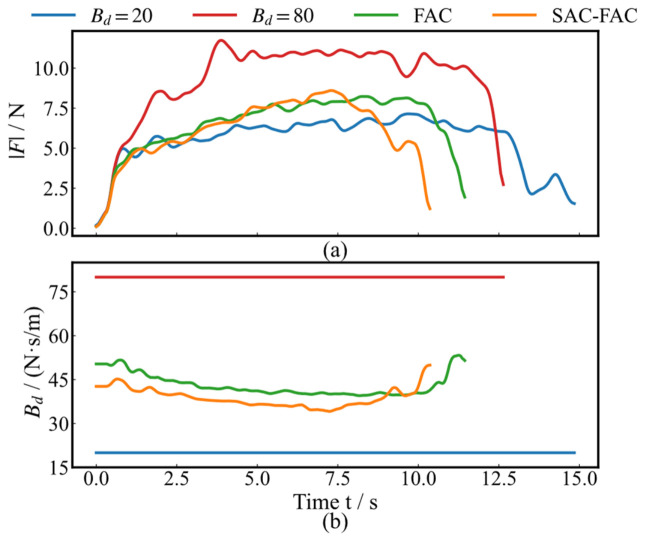
Time series of interaction force magnitude and damping coefficient during straight-line dragging: (**a**) interaction force; (**b**) damping coefficient.

Figure 11a indicates that the $B_{d}=80$ N·s/m strategy requires the largest interaction force, with a peak of approximately 12 N. The $B_{d}=20$ N·s/m strategy produces the lowest force magnitude but the longest dragging duration, approximately 14 s, which is consistent with its larger deviation in Figure 10a and indicates that low damping makes accurate trajectory control more difficult for the operator. The force magnitudes of FAC and SAC-FAC fall between the two fixed-damping strategies, with peaks of approximately 8 N; SAC-FAC’s peak is slightly lower than FAC’s.

Figure 11b shows that during the propulsion phase, the damping of FAC decreases from 50 N·s/m to approximately 40 N·s/m, while that of SAC-FAC decreases more substantially to approximately 33 N·s/m. Both recover toward 50 N·s/m near the end of the dragging process, indicating that SAC optimization preserves the damping-recovery behavior in the deceleration phase.

The quantitative metrics are summarized in Table 9.

As shown in Table 9, SAC-FAC yields a slightly lower mean $e_{RMSE}$ than FAC, decreasing from 3.11 mm to 3.04 mm. However, this reduction is not statistically significant (*p* = 0.518), indicating that the tracking-accuracy advantage of SAC-FAC is limited on the pure-propulsion straight-line trajectory. In contrast, SAC-FAC significantly reduces $W_{u}$ from 7.48 N to 6.99 N and $F_{RMS}$ from 6.97 N to 6.56 N, while increasing $I_{t}$ from 0.0138 to 0.0145 (*p* < 0.001). These results suggest that, for the straight-line task, the main benefit of SAC-FAC over FAC lies in reducing force-related cost and improving translational compliance rather than producing a statistically significant reduction in trajectory error. Compared with the two fixed-damping strategies, SAC-FAC achieves the lowest mean trajectory error, whereas the $B_{d}=20$ N·s/m strategy still provides lower force-related metrics and a higher $I_{t}$ at the cost of the largest trajectory error among the four strategies.

#### 4.3.2. Compound Trajectory

**Figure 12:**
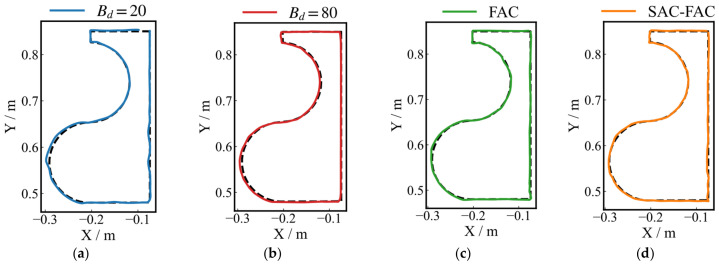
Compound dragging trajectories under four control strategies: (**a**) $B_{d}=20$ N·s/m; (**b**) $B_{d}=80$ N·s/m; (**c**) FAC; (**d**) SAC-FAC. Dashed lines: reference trajectory.

The compound trajectory tests controller response under frequent transitions between propulsion and turning. Figure 12 compares the dragging trajectories of the four strategies.

From Figure 12, the $B_{d}=20$ N·s/m strategy exhibits a noticeable deviation along the lower curved segment, indicating that low damping is insufficient for accurate directional control in turning phases. The $B_{d}=80$ N·s/m strategy follows the curved segment more closely, but a slight outward deviation remains along the right straight segment. FAC and SAC-FAC are closer to the reference trajectory overall, with SAC-FAC showing slightly tighter alignment along the curved segment.

**Figure 13:**
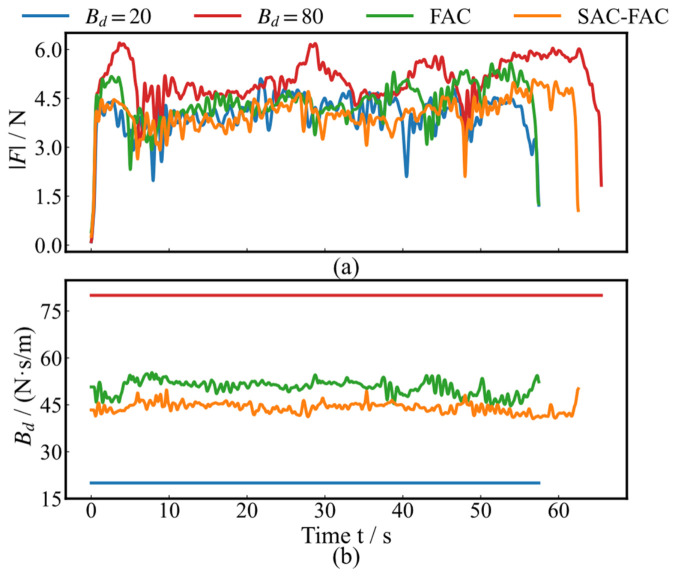
Time series of interaction force magnitude and damping coefficient during compound dragging: (**a**) interaction force; (**b**) damping coefficient.

Figure 13a indicates that the $B_{d}=80$ N·s/m strategy maintains the highest force level over most of the trial, mainly within the 5–6 N range. The $B_{d}=20$ N·s/m strategy and SAC-FAC show relatively low force magnitudes, predominantly within the 3–4.5 N range. This indicates that SAC-FAC reduces the interaction-force level relative to FAC while avoiding the higher force demand of the high-damping strategy.

Figure 13b shows that the damping of FAC fluctuates mainly between 45 and 55 N·s/m, whereas SAC-FAC operates approximately 3–5 N·s/m lower overall, mostly within the 43–48 N·s/m range. This indicates that the SAC-optimized controller maintains a generally lower damping level during compound dragging.

The quantitative metrics are summarized in Table 10.

SAC-FAC significantly improves the main performance metrics over FAC on the compound trajectory. The trajectory error $e_{RMSE}$ decreases from 2.64 mm to 1.67 mm, corresponding to a 36.7% reduction; $W_{u}$ decreases from 4.66 N to 4.43 N, corresponding to a 4.9% reduction; $F_{RMS}$ decreases from 4.51 N to 4.31 N, corresponding to a 4.4% reduction; and $I_{n}$ increases from 29.11 to 33.63, corresponding to a 15.5% improvement. These differences are statistically significant (*p* < 0.001). In contrast, $I_{t}$ remains nearly unchanged, increasing only from 0.0070 to 0.0071, and the difference is not statistically significant (*p* = 0.411).

The standard deviation of $e_{RMSE}$ also decreases from 1.20 mm for FAC to 0.25 mm for SAC-FAC, indicating more consistent trajectory tracking. Although the $B_{d}=20$ N·s/m strategy still yields lower force-related metrics and higher compliance indices, its trajectory error is markedly larger, 3.40 mm versus 1.67 mm. Overall, SAC-FAC provides a more balanced performance on the compound trajectory by improving trajectory accuracy and turning compliance while reducing force-related cost compared with FAC.

#### 4.3.3. 3D Ramp Trajectory

**Figure 14:**
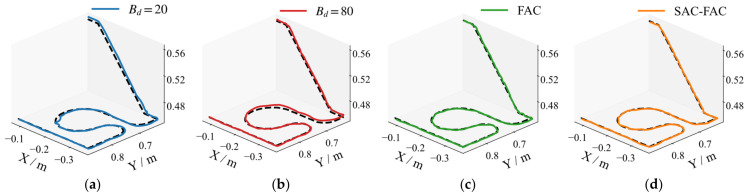
3D ramp dragging trajectories under four control strategies: (**a**) $B_{d}=20$ N·s/m; (**b**) $B_{d}=80$ N·s/m; (**c**) FAC; (**d**) SAC-FAC. Dashed lines: reference trajectory.

The 3D ramp trajectory tests how each strategy handles coupled horizontal path tracking and vertical force input. Figure 14 compares the dragging trajectories of the four strategies.

From Figure 14, the $B_{d}=20$ N·s/m strategy exhibits a noticeable inward deviation along the lower curved segment and lateral oscillation during the ascending phase of the ramp segment, indicating that low damping is insufficient for maintaining simultaneous path tracking and vertical-motion stabilization. The $B_{d}=80$ N·s/m strategy follows the ramp segment with moderate accuracy but shows visible deviations along the lower planar segments. Both FAC and SAC-FAC track the reference more closely than the two fixed-damping strategies, particularly along the lower straight and curved segments, but the difference between them is not visually distinguishable from the 3D plots alone and therefore requires quantitative comparison.

**Figure 15:**
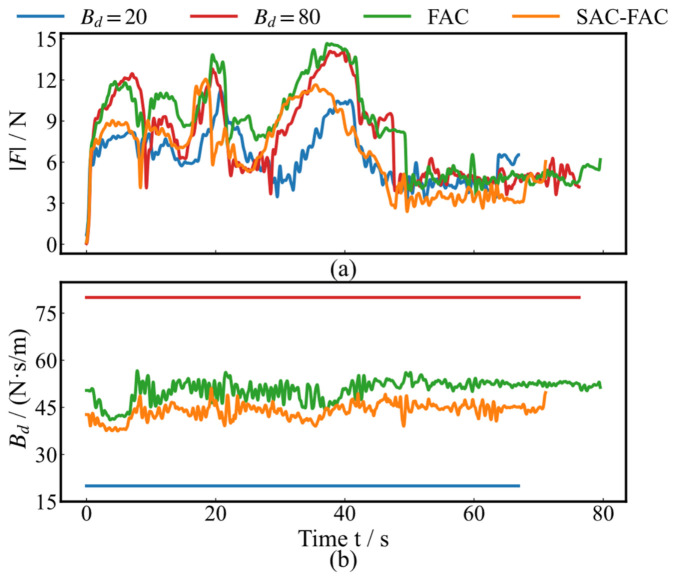
Time series of interaction force magnitude and damping coefficient during 3D ramp dragging: (**a**) interaction force; (**b**) damping coefficient.

Figure 15a shows that the force magnitudes are generally higher than those observed on the compound trajectory and remain elevated for most strategies relative to the straight-line case, which is consistent with the additional vertical force component required during the ramp segment. The force magnitudes of the $B_{d}=80$ N·s/m and FAC strategies repeatedly reach 12–14 N, with comparable force levels. The $B_{d}=20$ N·s/m strategy maintains a relatively low force range overall but with frequent fluctuations. SAC-FAC remains lower than the other three groups for most phases, particularly during the propulsion phases of 10–30 s and 50–65 s, where the force magnitude remains mostly within 5–8 N.

Figure 15b shows that the damping of FAC fluctuates mainly between 48 and 55 N·s/m, while SAC-FAC operates approximately 4–5 N·s/m lower overall, mostly within the 40–48 N·s/m range. During the 65–75 s interval at the end of dragging, the damping of both variable-admittance strategies recovers toward 50 N·s/m, indicating that the damping-recovery behavior is preserved after SAC optimization.

The quantitative metrics are summarized in Table 11.

SAC-FAC improves over FAC across all five metrics on the 3D ramp trajectory, and all FAC-versus-SAC-FAC differences are statistically significant (*p* < 0.001). The trajectory error $e_{RMSE}$ decreases from 3.45 mm to 2.76 mm, corresponding to a 20.0% reduction, while its standard deviation also decreases from 0.34 mm to 0.26 mm. $W_{u}$ decreases from 5.36 N to 4.11 N, corresponding to a 23.3% reduction, which is the largest improvement among the three trajectories. $F_{RMS}$ decreases from 8.20 N to 7.36 N, corresponding to a 10.2% reduction. In terms of compliance, $I_{t}$ increases from 0.0067 to 0.0086, corresponding to a 28.4% improvement, and $I_{n}$ increases from 23.46 to 25.26, corresponding to a 7.7% improvement.

However, the $B_{d}=20$ N·s/m strategy still yields better mean values than SAC-FAC in $F_{RMS}$, $I_{t}$, and $I_{n}$, although its $e_{RMSE}$ reaches 5.80 mm, which is the largest mean error reported in the three trajectory experiments. SAC-FAC’s $W_{u}$ is identical to that of the $B_{d}=20$ N·s/m strategy (both 4.11 N), but its $e_{RMSE}$ (2.76 mm) is substantially lower than the 5.80 mm of the $B_{d}=20$ N·s/m strategy. This indicates that SAC-FAC achieves much better spatial tracking accuracy while maintaining a comparable operator force cost.

Overall, across the three trajectories, SAC-FAC reduces the mean trajectory error and force-related metrics relative to FAC, with the most evident gains appearing on the compound and 3D ramp trajectories. The statistical results also show that the tracking-error improvement on the straight-line trajectory is limited, whereas the advantages of SAC-FAC become clearer as the task involves more frequent intent transitions, turning motion, and spatial coordination. The trajectory-dependent performance patterns are further discussed in Section 5.

## 5. Discussion

The real-robot experiments show that SAC-FAC reduced the mean $e_{RMSE}$, $W_{u}$, and $F_{RMS}$ by 19.5%, 11.6%, and 6.8%, respectively, relative to FAC. The statistical comparisons further indicate that these improvements are trajectory-dependent. On the straight-line trajectory, the reduction in $e_{RMSE}$ is small and not statistically significant, whereas the force-related metrics and $I_{t}$ are significantly improved. On the compound and 3D ramp trajectories, SAC-FAC shows clearer advantages in the main accuracy and force-related metrics, with all evaluated metrics significantly improved on the 3D ramp trajectory. The compliance indices also vary with trajectory geometry: $I_{t}$ mainly reflects propulsion performance in straight-line motion, whereas $I_{n}$ becomes more informative when curved or spatial motion is involved. This pattern suggests that SAC-optimized damping adaptation is more beneficial for tasks involving turning, braking, and vertical motion than for pure straight-line propulsion.

Although SAC was trained entirely on MuJoCo-synthesized force signals without real-robot data, the optimized parameters transferred to the physical platform without retuning for the tested tasks. This practical transfer stems in part from the episode-level optimization architecture. The scaling factors remain fixed within each training episode, and only fuzzy inference and admittance control are executed during online deployment. As a result, the SAC policy network is not directly transferred to the real robot, which helps mitigate the distribution-shift risk commonly associated with end-to-end policy transfer.

A direct cross-study numerical comparison with previous variable admittance studies [8,9,10,11] would be inconclusive because these works use different robot platforms, task protocols, and evaluation metrics. Hamad et al. [8] proposed an admittance-based force-scaling strategy, and Kang et al. [9] adjusted admittance behavior according to human-intention categories. These methods differ from SAC-FAC, in which the membership-function scaling factors are optimized offline rather than manually specified. Sharkawy et al. [10] introduced neural-network-based online adaptation of virtual inertia, which provides adaptive capability but offers less rule-level interpretability than fuzzy-rule deployment. Han et al. [11] proposed an energy-cost-based variable admittance controller with gradient-based parameter tuning, whereas SAC-FAC adopts fixed-parameter deployment after offline SAC optimization. During real-robot execution, the optimized fuzzy controller retains deterministic fuzzy inference and admittance control with an interpretable rule structure.

The ablation results further reveal a clear boundary-convergence tendency in the α-scaling factor. In G1, G3, and G4, where key training mechanisms were absent, $s_{\alpha }$ collapsed to the lower bound. In G5, the fixed curriculum schedule caused $s_{\alpha }$ to stall near its initial value because only limited training time remained at the highest disturbance level. Only the complete G2 configuration guided $s_{\alpha }$ toward an interior value. This behavior suggests that the present reward landscape strongly favors compression of the α membership functions unless PBRS, curriculum learning, and performance gating act together to counterbalance this tendency. Therefore, the main contribution of the complete training mechanism is not merely to improve the final reward, but to preserve the force-discrimination capability of the fuzzy controller while maintaining a deployable parameter configuration.

Several limitations should also be noted. First, all real-robot trials were conducted by a single operator; this design reduced operator-induced variability for controlled controller comparison, but it does not demonstrate robustness to inter-operator differences in strength, motion style, or experience. Second, the evaluation used objective trajectory- and force-related metrics, including trajectory tracking error, work per unit path, and RMS interaction force; these metrics quantify mechanical cost but do not directly measure subjective workload, fatigue, or perceived comfort. Third, sim-to-real transfer was evaluated only within the tested tasks, and the simulated force profiles cannot fully represent real human-operation variability. In addition, the optimizer comparison used five random seeds, so larger-scale seed analysis would further strengthen the characterization of optimizer stability. Finally, the current validation focused on Cartesian-position lead-through teaching with constrained orientation. Future work will include multi-operator experiments, subjective workload assessment, larger-scale random-seed analysis, and coupled position–orientation lead-through teaching.

## 6. Conclusions

SAC-FAC was developed as an offline-optimized fuzzy variable admittance control method for collaborative robot lead-through teaching. It pairs an interpretable fuzzy variable admittance controller, which maps operator intent to damping modulation, with offline SAC-based optimization of membership-function parameters. Three training mechanisms—a saturating task reward, potential-based reward shaping, and performance-gated curriculum learning—jointly stabilized the parameter search and mitigated parameter degradation under high-disturbance training. Across 600 lead-through trials covering three trajectories and four control strategies, SAC-FAC reduced $e_{RMSE}$, $W_{u}$, and $F_{RMS}$ by 19.5%, 11.6%, and 6.8% on average relative to the hand-tuned FAC, with corresponding improvements in $I_{t}$ and $I_{n}$ where applicable.

This offline-optimization–online-inference design decouples membership-function search from real-time control. Optimization is completed in simulation, whereas online deployment retains only deterministic fuzzy inference and admittance control, supporting deployable integration of deep reinforcement learning and interpretable fuzzy control. Future work will extend the proposed framework to coupled position–orientation lead-through teaching and broaden the validation through multi-operator experiments, subjective workload assessment, and larger-scale random-seed analysis.

## Figures and Tables

**Table 1:** Damping adjustment strategies for six representative dragging stages.

Dragging Stage	α Characteristic	β Characteristic	$B_{d}$ Adjustment
Stationary	α≈0	β≈0	Increase
Start-up	α≈0; small $\left|F_{\mathrm{ext}}\right|$	Small	Decrease
Acceleration	Large positive	Small	Decrease
Constant velocity	Nearly constant	Small	Maintain
Deceleration	α<0	Small	Increase
Turning	Small	Large	Increase

**Table 2:** Fuzzy rule base of the variable admittance controller.

$\Delta B_{d}$		β			
		ZO	S	M	B
α	NB	IL	IL	IL	IM
	NM	IL	IL	IM	IM
	NS	IM	IM	IM	IS
	ZO	KP	IS	IM	IL
	PS	DS	KP	IS	IM
	PM	DM	IS	IS	IM
	PB	DL	KP	IS	IS

**Table 3:** Disturbance levels and training force-amplitude ranges.

Index	η	Propulsion Force (N)	Lateral Force (N)	Braking Force (N)
0	0.50	[1.5, 7.5]	[0.75, 2.5]	[1.5, 5.0]
1	0.65	[1.95, 9.75]	[0.98, 3.25]	[1.95, 6.5]
2	0.80	[2.4, 12.0]	[1.2, 4.0]	[2.4, 8.0]
3	1.00	[3.0, 15.0]	[1.5, 5.0]	[3.0, 10.0]
4	1.20	[3.6, 18.0]	[1.8, 6.0]	[3.6, 12.0]

**Table 4:** SAC training hyperparameters.

Hyperparameter	Value
Policy network architecture	Two fully connected layers, 256 units each
Q-network architecture	Two fully connected layers, 256 units each
Learning rate	3 × 10^−4^
Discount factor γ	0.99
Soft update coefficient τ	0.005
Batch size	64
Replay buffer warm-up steps	100
Total training episodes	1000 (gated across 5 disturbance levels)

Note: The 1000 training episodes per seed are adaptively allocated across disturbance levels by performance gating, rather than evenly divided into 200 episodes per level.

**Table 5:** Comprehensive comparison of five optimization algorithms.

Algorithm	Mean Return (5 Seeds)	Best-Seed Return	Boundary Parameters ^1^	Training Time (s)	Training Budget ^2^
SAC	339.4 ± 10.9	350.7	7/15	1265	1000 ep
DDPG	329.9 ± 13.5	346.5	15/15	1167	1000 ep
TD3	329.1 ± 12.4	342.7	15/15	1188	1000 ep
PPO	339.5 ± 2.9	341.7	0/15	1175	1024 ts
BO	342.7 ± 3.5	347.9	2/15	28,796	25,000 env ep

^1^ Boundary-parameter definition and constraint ranges: see text. ^2^ ep = episodes; ts = timesteps; env ep = environment episodes. Standard deviations are sample standard deviations (ddof = 1). Training time denotes the wall-clock time of one training run under the stated budget.

**Table 6:** Local perturbation analysis of the five-seed best-return candidates.

Algorithm	Candidate Seed	Baseline Return ^1^	Max Return Increase	Observable Improvements ^2^	Feasible Directions ^3^
SAC	42	335.45	+0.23	0	4
DDPG	42	335.19	—	0	3
TD3	45	336.82	+0.16	0	3
PPO	45	330.58	+0.36	0	6
BO	43	334.01	+0.51	1	4

^1^ Baseline return before local perturbation. ^2^ Number of feasible directions with return increase > 0.5. ^3^ Number of feasible perturbation directions. “—” indicates no positive return increase.

**Table 7:** Ablation of training-mechanism components (“✓” = enabled, “—” = disabled).

Group	$r_{\mathrm{PBRS}}$	Curriculum	Performance Gating	Disturbance Schedule	$s_{\alpha }$	$s_{\beta }$	$s_{\Delta B}$	σ (last-50)
G1	—	—	—	Fixed η=1.0	0.9407	0.5055	1.5937	0.002
G2	✓	✓	✓	Gated, η: 0.5–1.2	0.9540	0.5080	1.5940	0.027
G3	—	✓	✓	Gated, η: 0.5–1.2	0.9405	0.5046	1.5947	0.0008
G4	✓	—	—	Fixed η=1.0	0.9407	0.5053	1.5937	0.002
G5	✓	✓	—	Fixed 200 ep/level, η: 0.5–1.2	0.9994	0.5063	1.5943	0.0007

**Table 8:** Configurations of the four control strategies.

Control Strategy	Damping Setting	Description
$B_{d}=20$ N·s/m	Fixed at 20 N·s/m	Low damping; high responsiveness
$B_{d}=80$ N·s/m	Fixed at 80 N·s/m	High damping; high stability
FAC	$B_{d0}=50$ N·s/m, $B_{d}\in \left[20,80\right]$ N·s/m	Hand-tuned fuzzy variable admittance
SAC-FAC	Same range as FAC	SAC-optimized fuzzy variable admittance

**Table 9:** Performance metrics of four control strategies on the straight-line trajectory (mean ± SD).

Control Strategy	$e_{\mathrm{RMSE}}$ (mm)	$W_{u}$ (N)	$F_{\mathrm{RMS}}$ (N)	$I_{t}$ (m/(N·s))
$B_{d}=20$ N·s/m	3.56 ± 0.80	6.61 ± 0.51	6.26 ± 0.42	0.0263 ± 0.0014
$B_{d}=80$ N·s/m	3.20 ± 0.28	11.17 ± 1.27	11.24 ± 0.96	0.0091 ± 0.0003
FAC	3.11 ± 0.23	7.48 ± 0.48	6.97 ± 0.39	0.0138 ± 0.0009
SAC-FAC	3.04 ± 0.67	6.99 ± 0.36	6.56 ± 0.29	0.0145 ± 0.0008
*p*-value (FAC vs. SAC-FAC)	0.518	<0.001	<0.001	<0.001

**Table 10:** Performance metrics of four control strategies on the compound trajectory (mean ± SD).

Control Strategy	$e_{\mathrm{RMSE}}$ (mm)	$W_{u}$ (N)	$F_{\mathrm{RMS}}$ (N)	$I_{t}$ (m/(N·s))	$I_{n}$ (rad/(N·m))
$B_{d}=20$ N·s/m	3.40 ± 0.17	4.17 ± 0.10	4.01 ± 0.10	0.0133 ± 0.0007	36.09 ± 5.95
$B_{d}=80$ N·s/m	2.77 ± 0.22	5.39 ± 0.27	5.23 ± 0.24	0.0053 ± 0.0003	22.54 ± 3.36
FAC	2.64 ± 1.20	4.66 ± 0.21	4.51 ± 0.20	0.0070 ± 0.0008	29.11 ± 5.02
SAC-FAC	1.67 ± 0.25	4.43 ± 0.11	4.31 ± 0.07	0.0071 ± 0.0003	33.63 ± 5.08
*p*-value (FAC vs. SAC-FAC)	<0.001	<0.001	<0.001	0.411	<0.001

**Table 11:** Performance metrics of four control strategies on the 3D ramp trajectory (mean ± SD).

Control Strategy	$e_{\mathrm{RMSE}}$ (mm)	$W_{u}$ (N)	$F_{\mathrm{RMS}}$ (N)	$I_{t}$ (m/(N·s))	$I_{n}$ (rad/(N·m))
$B_{d}=20$ N·s/m	5.80 ± 1.64	4.11 ± 0.24	6.77 ± 0.85	0.0114 ± 0.0010	28.99 ± 2.64
$B_{d}=80$ N·s/m	5.10 ± 1.10	6.72 ± 0.42	8.33 ± 0.75	0.0061 ± 0.0003	17.18 ± 0.80
FAC	3.45 ± 0.34	5.36 ± 0.33	8.20 ± 0.50	0.0067 ± 0.0004	23.46 ± 1.17
SAC-FAC	2.76 ± 0.26	4.11 ± 0.18	7.36 ± 0.33	0.0086 ± 0.0005	25.26 ± 1.25
*p*-value (FAC vs. SAC-FAC)	<0.001	<0.001	<0.001	<0.001	<0.001

## Data Availability

The data supporting the findings of this study are available from the authors upon reasonable request.
